# Measuring population health: association of self-rated health and PROMIS measures with social determinants of health in a cross-sectional survey of the US population

**DOI:** 10.1186/s12955-021-01854-1

**Published:** 2021-09-22

**Authors:** Janel Hanmer

**Affiliations:** Department of General Medicine, University of Pittsburgh, 230 McKee Place, Pittsburgh, PA 15213 USA

**Keywords:** Population health, Self-rated health, PROMIS, Social determinants of health, Health-related quality of life

## Abstract

**Background:**

Self-reported health-related quality of life is an important population health outcome, often assessed using a single question about self-rated health (SRH). The Patient Reported Outcomes Measurement Information System (PROMIS) is a new set of measures constructed using item response theory, so each item contains information about an underlying construct. This study’s objective is to assess the association between SRH and PROMIS scores and social determinants of health (SDoH) to evaluate the use of PROMIS for measuring population health.

**Methods:**

A cross sectional survey of 4142 US adults included demographics, 7 PROMIS domains with 2 items each, the PROMIS-preference (PROPr) score, self-rated health (SRH), 30 social determinants of health (SDoH), and 12 chronic medical conditions. SDoH and chronic condition impact estimates were created by regressing the outcome (PROMIS domain, PROPr, or SRH) on demographics and SDoH or a single chronic condition. Linear regression was used for PROMIS domains and PROPr; ordinal logistic regression was used for SRH.

**Results:**

Both SRH and PROPr detected statistically significant differences for 11 of 12 chronic conditions. Of the 30 SDoH, 19 statistically significant differences were found by SRH and 26 statistically significant differences by PROPr. The SDoH with statistically significant differences included those addressing education, income, financial insecurity, and social support. The number of statistically significant differences found for SDoH varies by individual PROMIS domains from 13 for Sleep Disturbance to 25 for Physical Function.

**Conclusions:**

SRH is a simple single question that provides information about health-related quality of life. The 14 item PROMIS measure used in this study detects more differences in health-related quality of life for social determinants of health than SRH. This manuscript illustrates the relative costs and benefits of each approach to measuring health-related quality of life.

## Background

There are a wide variety of indicators used to measure and monitor population health including mortality, disease prevalence, disability, and injury rates. Although these measures are critical, they do not capture health as perceived by the individuals within a population [1, 2]. Measures of health-related quality of life (HRQoL) provide a standardized survey-based approach to assess population health [3]. Indeed, a single-item global rating of HRQoL has been used in large US surveys since the 1940s [4]. The most widely used self-reported health (SRH) questions are slight variants of “In general, my health is: Excellent, Very Good, Good, Fair, or Poor”. [5] This item provides a general perception of health that reflects both objective health conditions and the individual’s values for different aspects of HRQoL. Multiple studies have found this question to be predictive of health care utilization and mortality [6–8].

HRQoL is an important outcome to monitor in population health. For example, in the United States, the Health People initiative sets data-driven national objectives to improve health and well-being over each decade and has included the Health Days and PROMIS-Global measures [9]. Models of population health include many factors beyond chronic conditions (CC) such as social determinants of health (SDoH) [2, 10, 11]. Therefore, any measures used to quality and monitor population health should be responsive to both CC and SDoH.

The measures selected for large surveys tended to be disease-agnostic (i.e., generic), providing an overall description of health not limited to one organ system or disease [12]. Because a single-item measure is a coarse method for measuring HRQoL, development of multiple-item generic HRQoL measures started in the 1970s with use in US national surveys by the early 2000s. For example, either the SF-36 or the VR-12 has been used in the Medicare Health Outcomes Survey since 1998 [13, 14], and the SF-12 has been in the Medical Panel Expenditures Survey since 2003 [15]. Despite the use of multi-item measures in many surveys, many other large US surveys of health, such as the National Health Interview Survey and the National Health and Nutrition Examination Survey, still rely on a single SRH item [16, 17]. The reliance on single-item measures is in part necessitated by many initiatives competing for limited space within these surveys. In addition, the argument for including multi-item generic HRQoL measures has been hampered by known problems such as ceiling effects in the general population, poorly worded questions, and licensing fees [18].

Recently, there have been significant advancements in generic HRQoL measures, including the development of the Patient-Reported Outcomes Measurement Information System (PROMIS), which is an initiative supported by the National Institutes of Health to create generic HRQoL measures using Item Response Theory (IRT) [19]. IRT is a psychometric method that calibrates a set of items on a construct (e.g., depression, pain, physical functioning) [20]. Any subset of items from the calibrated set can be used to get a score that is comparable to any other subset of items; the score from a clinical trial that measures depression using 8 items can be compared to the score from a population survey that measured depression using 2 items. There are currently over 90 adult health domains and over 20 pediatric health domains available through PROMIS [21]. There is also a PROMIS-Preference (PROPr) score that combines scores from 7 adult domains into a single preference-based summary score [22–24].

Several frameworks for understanding the relationships between SDoH, CC, medical care, and HRQoL are available [25]. This project assesses the sensitivity of SRH, PROMIS domains, and PROPr scores to SDoH and CC in a large US nationally representative sample to illustrate the relative costs and benefits of each measurement approach.

## Methods

This study is an extension of a previously published study using the same dataset, chronic conditions, and social determinates of health [26]. Briefly, the data are from a general population panel of US adults age 18 and older. The survey was offered in English and Spanish, both online and by phone. Participants completed several HRQoL questionnaires, self-reported 12 CC, and answered questions about 42 SDoH. This analysis uses 30 of the SDoH which are self-reported and excludes those linked by location (such as census tract information). For further details about the data and independent variables, please see the prior publication.

### Dependent variables

#### PROMIS domains

The survey included questions from 7 adult PROMIS domains: Cognitive Function—Abilities v2.0, Depression v1.0, Fatigue v1.0, Pain Interference v1.0, Physical Function v2.0, Sleep Disturbance v1.0, and Ability to Participate in Social Roles and Activities v2.0. The PROMIS questions refer to the participant’s own health “in the past 7 days” and have 5 response options. Participants answered 2 questions per domain for a total of 14 questions. Domains were scored by the scoring service on the Assessment Center, incorporating the default IRT parameters for each item [27]. PROMIS domains are scored such that the population mean is 50 with a standard deviation of 10. Higher scores represent more of the concept being measured; higher scores are better for the functional measures (cognitive function, physical function, ability to participate in social roles) and higher scores are worse for symptom measures (depression, fatigue, pain interference, sleep disturbance). In general, a minimally important difference (MID) for a PROMIS domain is between 3 and 5 points [28]; for this report, a difference of 4 points is considered to be a MID.

### PROPr

The PROPr scoring algorithm was developed for the 7 PROMIS domains collected in the survey [19–21]. The scoring was constructed using standard gamble valuations from a US sample of 983 adults. Possible PROPr scores range from − 0.022 (worst) to 1.0 (best) and the scale is anchored at the utility of dead (0) and the utility of full health (1.0). For this report, a difference of 0.04 is considered to be a MID [29].

#### Self-rated health (SRH)

This survey included the question, “In general, my health is: Excellent, Very Good, Good, Fair, or Poor” without a recall period. Data were coded such that Excellent = 1 and Poor = 5. Despite over 50 years of use, there is no established MID for SRH. Though any difference in response for an individual would be considered important, it is unclear what difference in a population is important.

### Analysis

CC and SDoH impact estimates were created by regressing the outcome (PROMIS domain, PROPr, or SRH) on nonmodifiable demographics (age, gender, race, ethnicity) and a single CC or SDoH as dummy variables. The coefficient(s) for the CC or SDoH is the estimated effect of having a condition/SDoH vs. not having it. Since the presence of disease was coded as higher and all SDoH were coded such that higher scores indicated more hardship, negative coefficients are expected for SRH, PROPr, and PROMIS functioning domains (cognitive function, physical function, and social roles); in contrast, positive coefficients are expected for PROMIS symptoms domains (depression, fatigue, pain interference, and sleep disturbance). Linear regression was used for PROMIS domains and PROPr as the PROMIS domains were IRT scored and utility measures are considered cardinal scales; ordinal logistic regression was used for SRH. A separate analysis was done for each CC and SDoH. Given the large number of models and coefficients, a coefficient was considered statistically significant if *p* < 0.001.

All analyses were performed using SAS 9.4 (The SAS Institute, Cary, NC). All analyses were weighted to be nationally representative. Ethics approval was given by [blinded] IRB PRO17080294.

## Results

**Fig. 1:**
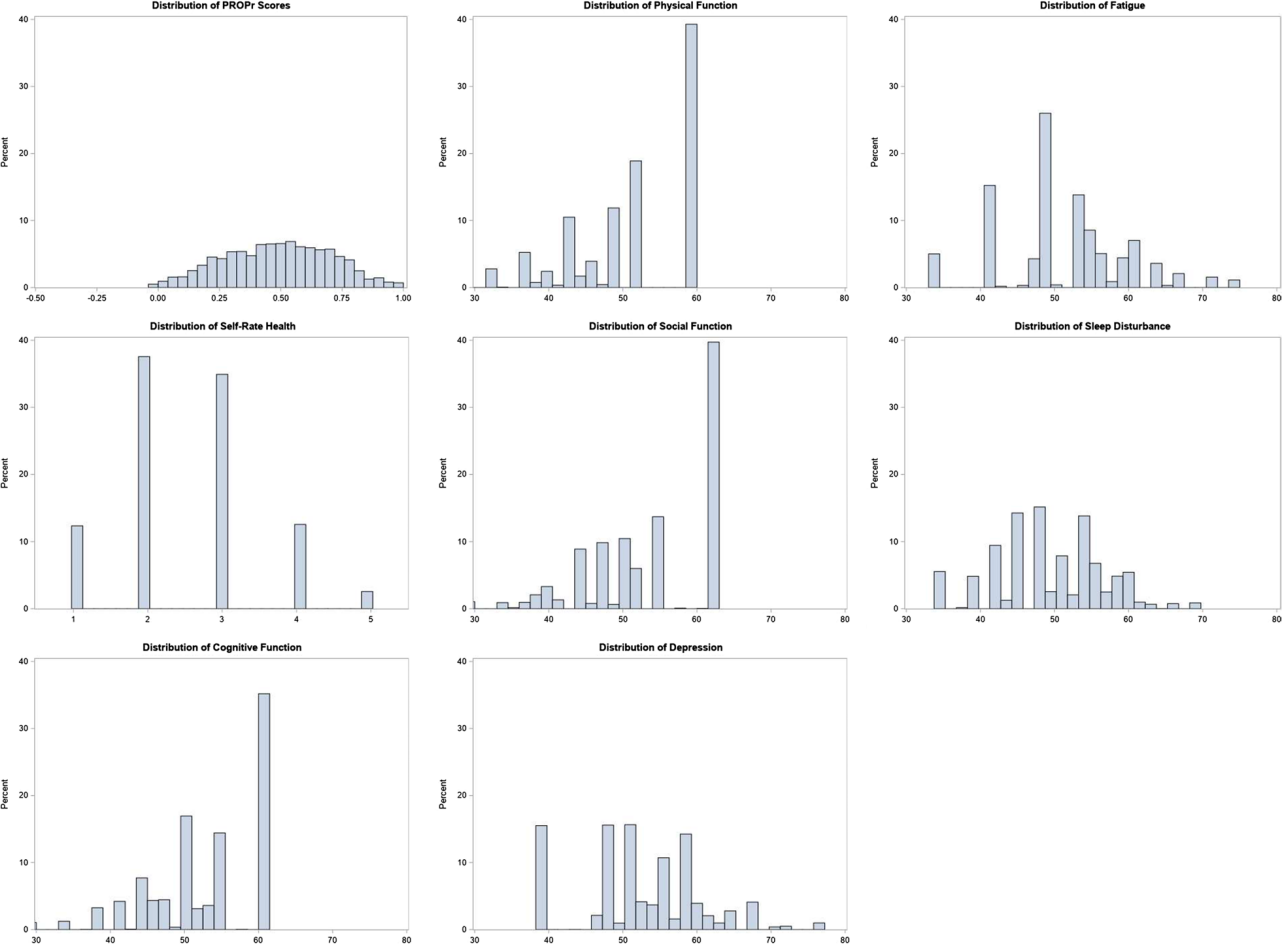
Distribution of each outcome

The sample had 4142 participants and full demographic details are available in the prior publication [26]. Negative SDoH exposures were common. For example, 45% reported difficulty paying their bills, 15% reported intimate partner violence within the last year, 30% reported some food insecurity, and 40% reported social isolation. Figure 1 illustrates the distribution of each outcome.

Table 1 includes the coefficients from all logistic and linear regression models. If a coefficient is statistically significant, it is italicized. If the coefficient is larger than the minimally important difference, it is bolded. All coefficients are in the expected direction (less than 1.0 for odds ratios, negative for PROPr and PROMIS function domains, positive for PROMIS symptom domains) except for “has a usual medical provider.” Of the statistically significant coefficients, both SRH and PROPr detect significant differences for 11 of the 12 CCs, but SRH only has statistically significant differences for 19 of the 30 SDoH whereas PROPr detects significant differences for 26 of them. Of the 37 statistically significant coefficients (both CC and SDoH) for PROPr, 36 reach MID; there is not an MID to apply to the 30 statistically significant odds ratios for SRH.

**Table 1:** Differences in scores associated with 12 chronic conditions and 30 social determinants of health for self-rated health, PROMIS-Preference, and 7 PROMIS domains

	Expect OR values < 1.0					Expect negative values of coefficients												Expect positive values of coefficients										
	OR SRH	Lower CI	Upper CI	*p*	PROPr	SE	p	Cog Func	SE	*P*	PhysFunc	SE	*P*	Soc roles	SE	*P*	Depr	SE	*P*	Fatig	SE	*P*	Pain Interf	SE	*P*	Sleep Dist	SE	*p*
Coronary heart disease	*0.379*	0.267	0.538	< .0001	***− 0.112***	0.020	< .0001	*− *2.2	0.8	0.0031	*− ****5.1***	0.9	< .0001	*− ****4.0***	0.8	< .0001	*2.9*	0.8	0.0002	*3.6*	0.7	< .0001	*3.5*	0.9	< .0001	1.6	0.7	0.0207
Angina	*0.324*	0.188	0.56	< .0001	*− ****0.191***	0.029	< .0001	*− ****4.3***	1.1	< .0001	*− ****8.0***	1.3	< .0001	*− ****6.0***	1.2	< .0001	***6.2***	1.1	< .0001	***6.2***	1.0	< .0001	***6.8***	1.2	< .0001	3.3	1.1	0.0045
Heart attack	*0.282*	0.18	0.441	< .0001	*− ****0.139***	0.024	< .0001	*− ****4.2***	0.9	< .0001	*− ****6.5***	1.1	< .0001	*− ****5.1***	1.0	< .0001	2.5	1.0	0.0106	***4.1***	0.8	< .0001	***5.0***	1.0	< .0001	1.8	0.8	0.0322
Stroke	*0.405*	0.255	0.643	< .0001	*− ****0.131***	0.024	< .0001	*− 3.2*	0.9	0.0004	*− ****6.6***	1.0	< .0001	*− ****4.0***	0.9	< .0001	2.4	1.1	0.0266	*3.5*	0.9	0.0002	*3.8*	1.1	0.001	0.4	0.9	0.6579
Emphysema	*0.21*	0.122	0.361	< .0001	*− ****0.230***	0.023	< .0001	*− *3.7	1.1	0.0011	*− ****9.2***	1.2	< .0001	*− ****7.9***	1.3	< .0001	***5.2***	1.3	< .0001	***4.9***	1.4	0.0003	***7.6***	1.2	< .0001	***5.0***	1.0	< .0001
Chronic obstructive pulmonary disease	*0.148*	0.103	0.212	< .0001	*− ****0.186***	0.018	< .0001	*− 3.6*	0.7	< .0001	*− ****8.2***	0.8	< .0001	*− ****7.1***	0.8	< .0001	***4.1***	0.8	< .0001	***5.5***	0.8	< .0001	***6.1***	0.8	< .0001	*3.0*	0.6	< .0001
Asthma	*0.546*	0.444	0.671	< .0001	*− ****0.095***	0.012	< .0001	*− 1.6*	0.4	0.0003	*− 3.3*	0.5	< .0001	*− 3.3*	0.5	< .0001	*2.4*	0.5	< .0001	*2.9*	0.4	< .0001	*3.2*	0.5	< .0001	*1.7*	0.4	< .0001
Cancer	0.853	0.675	1.078	0.1844	*− ***0.040**	0.014	0.0053	*− *0.9	0.5	0.0703	*− *1.4	0.6	0.0178	*− *1.3	0.5	0.0144	0.6	0.6	0.327	1.4	0.5	0.0079	1.1	0.6	0.0546	0.6	0.5	0.2412
Arthritis	*0.338*	0.281	0.406	< .0001	*− ****0.165***	0.010	< .0001	*− 2.8*	0.4	< .0001	*− ****7.2***	0.4	< .0001	*− ****5.5***	0.4	< .0001	*2.4*	0.4	< .0001	***4.4***	0.4	< .0001	***6.9***	0.4	< .0001	*3.1*	0.3	< .0001
Epilepsy	*0.339*	0.209	0.548	< .0001	*− ****0.181***	0.027	< .0001	*− ****6.1***	1.0	< .0001	*− ****6.7***	1.4	< .0001	*− ****5.0***	1.2	< .0001	3.3	1.3	0.0077	***4.1***	1.0	< .0001	***6.2***	1.2	< .0001	*2.8*	0.8	0.0007
Depression	*0.295*	0.246	0.354	< .0001	*− ****0.186***	0.009	< .0001	*− ****5.0***	0.3	< .0001	*− ****5.1***	0.4	< .0001	*− ****6.9***	0.4	< .0001	***6.2***	0.3	< .0001	***6.0***	0.4	< .0001	***6.0***	0.4	< .0001	*3.2*	0.3	< .0001
Diabetes	*0.277*	0.215	0.358	< .0001	*− ****0.122***	0.014	< .0001	*− 2.8*	0.6	< .0001	*− ****5.0***	0.6	< .0001	*− 3.8*	0.6	< .0001	*2.5*	0.6	< .0001	*3.2*	0.6	< .0001	***4.1***	0.6	< .0001	*1.6*	0.5	0.0005
Lowealth literacy	*0.654*	0.558	0.767	< .0001	*− ****0.104***	0.010	< .0001	*− 3.5*	0.3	< .0001	*− 3.9*	0.4	< .0001	*− 3.7*	0.4	< .0001	*2.5*	0.4	< .0001	*2.4*	0.4	< .0001	*3.6*	0.4	< .0001	0.9	0.3	0.0048
Education less than high school (reference: college)	*0.248*	0.177	0.349	< .0001	*− ****0.171***	0.020	< .0001	*− ****5.1***	0.7	< .0001	*− ****6.4***	0.8	< .0001	*− ****4.9***	0.8	< .0001	*3.3*	0.7	< .0001	*3.5*	0.7	< .0001	***5.8***	0.9	< .0001	2.0	0.6	0.0023
Education high school (reference: college)	0.445	0.365	0.541	0.2243	*− ****0.076***	0.011	< .0001	*− 2.3*	0.4	< .0001	*− 3.6*	0.4	< .0001	*− 2.1*	0.4	< .0001	1.3	0.4	0.0021	1.2	0.4	0.0047	*2.9*	0.4	< .0001	0.9	0.4	0.0145
Education some college (reference: college)	0.509	0.435	0.595	0.4756	*− ****0.084***	0.009	< .0001	*− 2.3*	0.3	< .0001	*− 2.8*	0.3	< .0001	*− 2.5*	0.3	< .0001	*1.9*	0.3	< .0001	*2.1*	0.3	< .0001	*3.0*	0.4	< .0001	*1.8*	0.3	< .0001
Nousual medical provider	1.257	1.034	1.529	0.0217	0.016	0.012	0.1619	0.3	0.4	0.5076	1.3	0.5	0.0058	0.4	0.4	0.3747	0.3	0.4	0.5067	*− *0.9	0.4	0.0256	*− *0.8	0.5	0.0751	*− *0.5	0.4	0.1604
Low medical provider CAHPS scores	*0.688*	0.575	0.824	< .0001	*− ****0.078***	0.010	< .0001	*− 3.1*	0.4	< .0001	*− 1.4*	0.4	0.0006	*− 2.7*	0.4	< .0001	*2.5*	0.4	< .0001	*1.9*	0.4	< .0001	*1.8*	0.4	< .0001	*1.6*	0.3	< .0001
Difficulty getting to medical appointments	*0.366*	0.278	0.482	< .0001	*− ****0.213***	0.013	< .0001	*− ****6.5***	0.5	< .0001	*− ****7.7***	0.6	< .0001	*− ****7.6***	0.7	< .0001	***5.4***	0.5	< .0001	***5.6***	0.5	< .0001	***8.0***	0.6	< .0001	*3.3*	0.5	< .0001
Medical insurance—uninsured (reference: commercial)	0.6	0.468	0.769	0.5507	*− ****0.071***	0.015	< .0001	*− 2.0*	0.5	0.0002	*− 2.2*	0.6	0.0002	*− 2.4*	0.5	< .0001	1.6	0.6	0.0037	1.1	0.5	0.0428	*2.5*	0.5	< .0001	1.2	0.5	0.0095
Medical insurance—Medicare (reference: commercial)	0.518	0.42	0.641	0.0267	*− ****0.122***	0.013	< .0001	*− 3.3*	0.4	< .0001	*− ****5.4***	0.5	< .0001	*− 3.5*	0.5	< .0001	*2.0*	0.5	< .0001	*2.8*	0.4	< .0001	***4.3***	0.5	< .0001	1.3	0.4	0.0014
Medical insurance—Medicaid (reference: commercial)	*0.412*	0.31	0.547	0.0002	*− ****0.106***	0.017	< .0001	*− 3.2*	0.6	< .0001	*− ****4.5***	0.7	< .0001	*− 3.5*	0.7	< .0001	2.0	0.7	0.0017	1.9	0.7	0.0076	*3.9*	0.7	< .0001	*2.4*	0.5	< .0001
Medical insurance—other (reference: commercial)	0.831	0.577	1.198	0.0777	*− *0.044	0.020	0.0266	*− *1.7	0.7	0.0217	*− *1.0	0.9	0.2615	*− *1.1	0.8	0.1659	1.1	0.7	0.1399	0.8	0.7	0.2936	*2.4*	0.7	0.0004	*− *0.3	0.7	0.7152
Difficulty paying medical deductibles	*0.515*	0.436	0.607	< .0001	*− ****0.087***	0.010	< .0001	*− 2.6*	0.3	< .0001	*− 2.7*	0.4	< .0001	*− 2.9*	0.4	< .0001	*1.9*	0.4	< .0001	*2.3*	0.4	< .0001	*3.2*	0.4	< .0001	*2.3*	0.3	< .0001
Difficulty paying medical copays	*0.408*	0.339	0.491	< .0001	*− ****0.132***	0.011	< .0001	*− 3.7*	0.4	< .0001	*− ****4.5***	0.4	< .0001	*− ****4.2***	0.4	< .0001	*2.9*	0.4	< .0001	*3.1*	0.4	< .0001	***4.9***	0.4	< .0001	*3.1*	0.3	< .0001
Difficulty paying monthly bills	*0.384*	0.329	0.448	< .0001	*− ****0.131***	0.009	< .0001	*− 3.8*	0.3	< .0001	*− 3.8*	0.4	< .0001	*− ****4.4***	0.3	< .0001	*3.2*	0.3	< .0001	*3.5*	0.3	< .0001	***4.3***	0.4	< .0001	*3.3*	0.3	< .0001
Experienced intimate partner violence in the last year	*0.634*	0.516	0.779	< .0001	*− ****0.145***	0.011	< .0001	*− ****4.2***	0.4	< .0001	*− 3.6*	0.5	< .0001	*− ****5.2***	0.5	< .0001	***4.3***	0.4	< .0001	***4.3***	0.4	< .0001	***5.3***	0.5	< .0001	*2.4*	0.4	< .0001
Food insecurity	*0.279*	0.232	0.336	< .0001	*− ****0.208***	0.010	< .0001	*− ****5.7***	0.4	< .0001	*− ****6.5***	0.4	< .0001	*− ****6.9***	0.4	< .0001	***5.1***	0.4	< .0001	***5.5***	0.4	< .0001	***7.0***	0.4	< .0001	***4.1***	0.3	< .0001
Social isolation	*0.753*	0.647	0.876	0.0002	*− ****0.056***	0.009	< .0001	*− 1.6*	0.3	< .0001	*− 1.4*	0.4	0.0002	*− 2.1*	0.4	< .0001	*1.9*	0.3	< .0001	*1.9*	0.3	< .0001	*1.4*	0.4	0.0002	*1.7*	0.3	< .0001
Stress	*0.333*	0.271	0.409	< .0001	*− ****0.241***	0.010	< .0001	*− ****5.9***	0.4	< .0001	*− ****5.4***	0.5	< .0001	*− ****8.3***	0.4	< .0001	***7.8***	0.4	< .0001	***7.7***	0.4	< .0001	***7.4***	0.5	< .0001	***5.8***	0.3	< .0001
Low social support	*0.384*	0.311	0.474	< .0001	*− ****0.136***	0.012	< .0001	*− 3.7*	0.4	< .0001	*− 3.2*	0.5	< .0001	*− ****4.7***	0.5	< .0001	***4.5***	0.5	< .0001	*3.2*	0.5	< .0001	*3.9*	0.5	< .0001	*3.3*	0.4	< .0001
Living without a partner	0.802	0.691	0.931	0.0038	*− 0.034*	0.009	0.0003	*− *0.5	0.3	0.1085	*− 1.4*	0.4	0.0001	*− 1.4*	0.4	< .0001	1.1	0.3	0.0018	0.9	0.3	0.0056	0.9	0.4	0.0194	*− *0.3	0.3	0.2795
Employment–self (reference: employed)	*1.117*	0.841	1.485	< .0001	*− *0.004	0.016	0.788	*− *1.1	0.6	0.0575	*− *0.4	0.6	0.4348	*− *0.2	0.6	0.6906	*− *0.2	0.6	0.7506	0.0	0.6	0.9786	1.0	0.6	0.109	*− *1.1	0.5	0.0373
Employment—looking for work (reference: employed)	0.656	0.454	0.947	0.4948	*− ****0.091***	0.022	< .0001	*− 3.1*	0.8	< .0001	*− *2.4	0.9	0.0048	*− ****4.3***	0.9	< .0001	2.1	0.8	0.0115	1.0	0.8	0.2145	2.6	0.8	0.0016	1.1	0.7	0.1089
Employment—retired (reference: employed)	0.752	0.581	0.973	0.0295	*− ****0.058***	0.014	< .0001	*− 1.7*	0.5	0.0007	*− ****4.1***	0.6	< .0001	*− *1.8	0.6	0.0015	*− *0.2	0.5	0.7151	0.6	0.5	0.241	*1.9*	0.6	0.0008	*− *1.0	0.5	0.0262
Employment—disabled (reference: employed)	*0.1*	0.073	0.137	< .0001	*− ****0.280***	0.015	< .0001	*− ****7.5***	0.6	< .0001	*− ****13.1***	0.6	< .0001	*− ****10.3***	0.6	< .0001	***4.7***	0.7	< .0001	***7.1***	0.6	< .0001	***10.4***	0.6	< .0001	*3.0*	0.5	< .0001
Employment—other (reference: employed)	0.749	0.561	1.001	0.0556	*− *0.020	0.017	0.2501	*− *1.2	0.6	0.0683	*− *1.8	0.6	0.0039	*− *1.0	0.7	0.148	0.6	0.6	0.3715	0.4	0.6	0.5247	0.4	0.7	0.6047	*− *0.5	0.6	0.3749
Household income $60–100 k (reference: > 100 k)	*0.666*	0.536	0.827	< .0001	*− *0.035	0.012	0.0035	*− *1.0	0.4	0.0235	*− 1.6*	0.5	0.0006	*− *1.5	0.5	0.0022	1.0	0.5	0.0251	0.9	0.4	0.0522	0.9	0.5	0.078	0.5	0.4	0.2694
Household income $40–60 k (reference: > 100 k)	0.578	0.457	0.731	0.0568	*− ****0.056***	0.013	< .0001	*− *1.3	0.5	0.0039	*− 3.0*	0.5	< .0001	*− 2.1*	0.5	< .0001	1.3	0.5	0.0125	1.0	0.5	0.0462	1.0	0.5	0.0431	0.9	0.4	0.0309
Household income $20*–*40 k (reference: > 100 k)	*0.382*	0.304	0.48	0.0002	*− ****0.101***	0.013	< .0001	*− 2.9*	0.5	< .0001	*− ****4.5***	0.5	< .0001	*− 3.2*	0.5	< .0001	*2.1*	0.5	< .0001	*2.1*	0.5	< .0001	*2.8*	0.5	< .0001	1.2	0.4	0.0052
Household income < $20 k (reference: > 100 k)	*0.213*	0.163	0.277	< .0001	*− ****0.141***	0.016	< .0001	*− 3.8*	0.5	< .0001	*− ****6.4***	0.6	< .0001	*− ****4.4***	0.6	< .0001	*3.1*	0.6	< .0001	*3.0*	0.6	< .0001	***4.5***	0.6	< .0001	1.2	0.5	0.0194
No house ownership	*0.646*	0.547	0.762	< .0001	*− ****0.048***	0.010	< .0001	*− 1.5*	0.4	< .0001	*− 2.0*	0.4	< .0001	*− 1.4*	0.4	0.0002	1.0	0.4	0.0071	1.2	0.4	0.0023	1.1	0.4	0.0073	0.5	0.3	0.1225
Total statistically significant	30				37			27			36			34			25			27			32			20		
Total significant that are > MID	N/A				36			9			21			20			10			12			18			3		

Coefficients are italicized, if the *p* value is < 0.001. Coefficients are bolded, if they reach a minimally important difference

CI, confidence interval; Cog Func, cognitive function; Depr, depression; Fatig, fatigue; OR, odds ratio; Pain Interf, pain interference; Phys Func, physical function; PROPr, PROMIS-preference; Sleep Dist, sleep disturbance; Soc Roles, social roles; SRH, self-rated health

As an illustration of the use of different measures, consider the results for food insecurity, which is a good exemplar of SRH changes because it has one of the lowest odds ratios (0.279) and has received a full exploration in a prior publication [30]. Food insecurity was assessed based on responses to 3 food insecurity items used in the USDA Household Food Security Survey Module [31]. The items ask how often, in the last 12 months, the respondent or people in the respondent’s household (1) worried whether your food would run out before you had money to buy more; (2) the food that you bought did not last, and you didn’t have enough money to get more; or (3) you couldn’t afford to eat balanced meals. Response options were “Always,” “Usually,” “Sometimes,” “Rarely,” and “Never.” Participants who responded “Always” or “Usually” to any of these questions were categorized as food insecure. Adjusting for survey weights, 14.1% of respondents were food insecure.

Table 2 shows the proportion of SRH responses for a 47-year-old non-Hispanic white female (the average respondent in the sample) using the logistic regression results. The table illustrates the distribution of responses in the entire sample and in the hypothetical case. The logistic regression estimates that food-secure 47-year-old non-Hispanic white females, when compared to food-insecure 47-year-old non-Hispanic white females, are more likely to report “excellent” or “very good” health (58.0% vs 28.3%). These estimated distributions are dependent on the other covariates (age, gender, race, ethnicity) entered in the logistic regression results.

**Table 2:** Distribution of self-rated health responses in the entire sample and two hypothetical cases

	Whole sample			Estimated responses for a 47-year-old non-Hispanic white female	
	Unweighted frequency	Weighted frequency	Weighted percent	Food secure percent	Food insecure percent
Excellent	477	512	12.4	14.5	4.6
Very good	1543	1555	37.6	43.5	23.7
Good	1511	1446	34.9	32.5	45.1
Fair	499	521	12.6	7.9	21.5
Poor	109	107	2.6	1.5	5.1

In comparison, the coefficient for food insecurity when the outcome is PROPr is − 0.208. This estimate is 5 times the size of the assumed minimally important difference for PROPr, nearly a standard deviation of PROPr in this sample (0.215), and over 20% of PROPr’s range—a difference larger than those any CC except emphysema. PROPr, by definition, also provides access to 7 PROMIS domain scores. For food insecurity, all PROMIS domains show differences that are both statistically significant and are larger than the MID. The largest coefficients are for Pain Interference (7.0) and Social Roles (− 6.9) which is a difference of 0.7 standard deviations and a difference larger than those seen in most of the CCs.

Table 1 shows also that the number of significant coefficients varies by PROMIS domain. The number of coefficients that are both statistically significant and reach the MID are 3 for Sleep Disturbance, 9 for Cognitive Function, 10 for Depression, 12 for Fatigue, 18 for Pain Interference, 20 for Social Roles, and 21 for Physical Function.

## Discussion

This manuscript compares the costs and benefits of using a single SRH question compared to 14-questions from PROMIS that cover 7 health domains. Though it may be obvious that using more questions provides more information [32], the composite score for PROMIS was different, to a statistically significant degree, for 37 of the tested CCs and SDoH whereas SRH was different, to a statistically significant degree, for 30. More importantly, the additional questions improve interpretability of the analyses. SRH requires analytical techniques for ordinal outcomes whose results are generally difficult to interpret (e.g., odds ratios or relative risk ratios) [33]; in contrast, continuous outcomes such as PROMIS and PROPr allow analytical techniques with results that can be described as differences on a numeric scale. The example used in this report is that food insecurity is associated with an odds ratio of 0.279 for being in a better category of SRH and associated with a difference of − 0.208 on the PROPr scale (which is constructed such that 0 is equivalent to the utility of “dead” and 1 is the utility of “full health”). Changes in PROPr score can be evaluated using PROPr’s MID (0.04), standard deviation (0.215), or range (− 0.022 to 1.0). This difference in interpretability is important as health care and public health have placed increasing emphasis on person-centered outcomes such as health-related quality of life and well-being [34, 35].

Though PROPr detected more statistically significant differences than SRH overall, SRH had statistically significant findings in 3 SDoH where PROPr did not. For one of these, income of $60,000–100,000 when compared to income of over $100,000, PROPr was in the same expected direction as SRH but did not reach the strict statistical significance used in this analysis. Likewise, both PROPr and SRH indicated better HRQoL for those who did not have a usual medical provider, though only SRH was statistically significant. Though the initial expectation was that respondents without a usual medical provider would have worse HRQoL, it may be that people with health conditions are more likely to have a provider than those who are healthier. The SDoH where SRH and PROPr diverge in direction is for self-employed individuals compared to employed individuals. SRH has a statistically significant finding of better HRQoL in this group where PROPr has a nonsignificant finding towards worse HRQoL. Assuming that this result is not spurious, it may be that people who are self-employed have a better global view of their overall HRQoL, as measured by SRH, but are not different from the general population when asked more specific questions about their symptoms and function, as measured by PROPr. Within the PROMIS domains, those who are self-employed do not have statistically significantly different scores than those who are employed, though they have a trend towards better sleep.

The PROMIS domains with the most statistically significant coefficients that reached the MID threshold were physical functioning, social functioning, and pain interference. With only 2 excpetions, within any CC or SDoH, no other PROMIS domain reach the MID threshold without these 3 domains also reaching the MID threshold. The first exception is respondents who reported experiencing interpersonal violence within the last year. The difference in physical functioning does not reach the MID threshold, but cognitive function, social function, depression, fatigue, and pain interference do. The second exception is respondents reporting low social support. Neither physical functioning or pain interference reach the MID threshold, but social function and depression do.Many health-related quality of life measures exist [4] and some have been used for monitoring the health of populations [13–15]. PROMIS is an exciting advancement in HRQoL measurement because it is based on Item Response Theory (IRT) rather than Classical Test Theory. IRT is a modern measurement technique with a long history in educational testing. It calibrates a large number of items on a concept (such as pain or depression) to make an “item bank.” Scores using any subset of the item bank can be compared to scores using any other subset of the item bank as long as they use the same calibration parameters. While only using 2 items per PROMIS domain creates scores with low reliability, making it too coarse to track individuals across time, these scores are appropriate for group-level analyses. Furthermore, PROMIS scores collected using any number of items are commensurable with scores derived from studies which collected the same domains using a different number of (and possibly altogether different) questions. Finally, in contrast to many other HRQoL measures, PROMIS is free to use in English and Spanish.

Summary scores of HRQoL can be constructed using psychometric techniques or econometric techniques [32]. Psychometric techniques such as factor analysis usually result in 2 or 3 summary scores, such as the mental and physical health summary scores for the PROMIS-Global and PROMIS-29 [36, 37]. In contrast, health utility measures have a single summary score that estimates the value of HRQoL. Health utility measures are most often designed for economic analyses such as cost-effectiveness analysis where they are used to estimate quality-adjusted life years (QALYs) [38]. Health utility scores are constructed to represent the average preferences of a population such as the adult population within a country. This construction makes them appropriate for policy and resource allocation decisions that impact the entire population. Health utility scores, therefore, are not appropriate for individual level decision making because the average preferences of a population do not necessarily reflect the preferences of an individual, much like how an individual can prefer a particular political candidate who did not win an election. Because health utility scores represent the preferences of a population make these scores good candidates for monitoring population health because they measure both the amount and the value of HRQoL in a single number.

The differences associated with CCs and SDoH in this report are adjusted for unmodifiable demographic factors (age, gender, race, ethnicity). A full understanding of these CCs and SDoH would require further adjustments that are informed by theoretical models for each CC and SDoH. For example, models of food insecurity should be adjusted for other correlated factors such as household income [30]; each row of Table 1 could have a full independent exploration and the data are available for such analyses. As such, comparisons across or within CCs or SDoH should be interpreted as illustrations of the approach and not as well-developed estimates of differences for use in policy decisions. This manuscript is meant to illustrate the value of multiple subscales with a composite score which can provide a better understanding of the complexity of a population’s experience.

Fourteen questions represent a substantial survey response burden when compared to a single question.This survey burden is the primary cost of including extra questions to measure HRQoL. Survey researchers often recommend keeping surveys less than 15 min long and a general rule-of-thumb is that each question in a survey takes 6 seconds (though this varies by survey mode, question characteristics, and respondent characteristics) [39–41]. However, the use of PROMIS may allow for thoughtful substitutions of other measures within a larger survey. One benefit of IRT-based measures is the ability to “co-calibrate” with other measures. This technique calibrates the items from a measure onto an appropriate item bank; for example, the Kessler 6 has been calibrated onto the PROMIS Depression item bank [42]. A library of these co-calibrations can be found at at PROsetta stone [43]. This technique both shows that the measures are capturing the same construct and provides look-up tables to convert one score to another score. These efforts can bring data from disparate sources onto the same metric, even allowing a longitudinal survey to change its measures in order to gain additional psychometric information while not sacrificing commensurability with earlier data.

This study should be interpreted in light of several limitations. Since the data were cross-sectional, it is unknown if the differences seen in this study have a causal relationship or correspond to changes over time. The analyses of each CC and SDoH were standardized and therefore may not be appropriately adjusted based on theoretical models, so the relative impact across CC and SDoH should be interpreted with caution. Finally, online panel surveys can be biased with regard to which individuals participate. However, this particular panel uses face-to-face recruitment to help mitigate this concern, and weighting to help account for non-response bias. These limitations are balanced by several strengths. Data for this study came from a large nationally representative survey, had a high response rate, and participants answered questions about a wide range of SDoH using questions from other national surveys.

## Conclusions

Person-centered outcomes are increasingly important in clinical practice, research, and public health. There has been a commensurate improvement in person-centered outcome measurement, particularly with the development of measures constructed using IRT. IRT-based measures allow flexible administration and a common metric upon which to compare legacy measures. IRT-calibrated questions can give a substantial amount of information about the construct they are measuring; this study uses just 2 questions per HRQoL domain and shows sensitivity to a variety of SDoH. Cross-cutting IRT measures such as PROMIS can be used to measure HRQoL in any disease process; this manuscript provides evidence that they are also sensitive to SDoH, making them appropriate for use in public health measurement and monitoring.

Single-item SRH has had a long history as a measure of HRQoL. Its strengths include its ubiquity and its brevity. However, as focus shifts to person-centered outcomes like HRQoL, SRH has several limitations as an outcome, including results that are difficult to interpret and that are less sensitive to SDoH than those derived from longer measures. This manuscript illustrates the difficulties in using SRH as an outcome measure when compared to continuous outcome measures such as PROMIS and PROPr. While there is always a cost to adding more questions to surveys, the depth of information provided by IRT-based HRQoL measures may justify those costs.

## Data Availability

The datasets generated and/or analysed during the current report are available in the OSF repository, https://osf.io/63548/.

## Abbreviations

CC: Chronic conditions
HRQoL: Health-related quality of life
IRT: Item response theory
MID: Minimally important difference
PROMIS: Patient-reported outcomes measurement information system
PROPr: PROMIS-preference
SDoH: Social determinants of health
SRH: Self-reported health
